# Macrophage plasticity and function in cancer and pregnancy

**DOI:** 10.3389/fimmu.2023.1333549

**Published:** 2024-01-11

**Authors:** Tingxuan Yin, Xinyi Li, Yanhong Li, Xingxing Zang, Lu Liu, Meirong Du

**Affiliations:** ^1^ Lab of Reproduction Immunology, Shanghai Key Laboratory of Female Reproductive Endocrine-Related Diseases, Hospital of Obstetrics and Gynecology, Fudan University Shanghai Medical College, Shanghai, China; ^2^ Department of Microbiology and Immunology, Albert Einstein College of Medicine, Bronx, NY, United States

**Keywords:** macrophages, plasticity, maternal-fetal interface, tumor-associated macrophages (TAMs), immunosuppression, immune microenvironment

## Abstract

As the soil of life, the composition and shaping process of the immune microenvironment of the uterus is worth exploring. Macrophages, indispensable constituents of the innate immune system, are essential mediators of inflammation and tissue remodeling as well. Recent insights into the heterogeneity of macrophage subpopulations have renewed interest in their functional diversity in both physiological and pathological settings. Macrophages display remarkable plasticity and switch from one phenotype to another. Intrinsic plasticity enables tissue macrophages to perform a variety of functions in response to changing tissue contexts, such as cancer and pregnancy. The remarkable diversity and plasticity make macrophages particularly intriguing cells given their dichotomous role in either attacking or protecting tumors and semi-allogeneic fetuses, which of both are characterized functionally by immunomodulation and neovascularization. Here, we reviewed and compared novel perspectives on macrophage biology of these two settings, including origin, phenotype, differentiation, and essential roles in corresponding microenvironments, as informed by recent studies on the heterogeneity of macrophage identity and function, as well as their mechanisms that might offer opportunities for new therapeutic strategies on malignancy and pregnancy complications.

## 1 Introduction

Macrophages are a subset of mononuclear phagocytes of the innate immune system. As a multifunctional immune population, they not only play a central role in inflammation and host defense but also exert homeostatic functions in a wide range of peripheral tissues. Remarkably, macrophages are one of the most plastic and diverse cells among human hematopoietic cells, involved in nearly all diseases in humans. In local tissues, microenvironmental clues mold macrophages morphologically and functionally according to the homeostatic needs of their surroundings, leaving a profound imprint on the tissue-specific functions of macrophages.

As a place where the embryo implants and develops, the maternal–fetal interface is highly dynamic and multi-functional to maintain a homeostatic environment that requires both immunotolerance toward the semi-allogeneic fetus, defense against pathogens, and tissue remodeling. At the maternal–fetal interface, macrophages are present in the whole stage of gestation, where they participate in various biological processes dynamically, including decidualization, trophoblast invasion, angiogenesis, parturition, and postpartum uterine involution (1). Similarly, in the setting of cancer, phenotype shift and function skewing of macrophages also occur along with tumor initiation, progression, and metastasis (2). Originally, activated macrophages central to the chronic inflammatory process mediate the elimination of senescent epithelial cells and tumor tissue disruption, which drives subsequent carcinogenesis (3). Later upon tumor establishment, cancer cell products transform neighboring macrophages from a classically activated state to a trophic, immunoregulatory, and pro-angiogenic phenotype that favors tumor escape (4).

Dates back 30 years ago, some researchers noticed that the penetrative property of hemochorial placentation mimics the phenomena observed in highly invasive tumors, and both are accompanied by a hypoxic environment and abundant immune cells (5, 6). Indeed, as a kind of versatile immune component, macrophages contribute a lot to the formation of immune tolerance and new blood vessels through their extreme plasticity and diversity (7). However, investigations that draw parallels between the two are lacking (5, 7, 8). Recent studies combining lineage tracing, single-cell multi-omics, and morphologic and molecular analysis have illustrated the essential role and mechanisms of diverse macrophage populations in mediating various aspects of both pathological processes like tumor development and physiological status as pregnancy (9–11). In this review, we described the phenotypical heterogeneity and factors regulating macrophage differentiation in the context of both cancer and pregnancy and compared the concrete mechanisms of how macrophages exert immune-modulatory and angiogenic capacities.

## 2 The microenvironment of tumor and decidua

The maternal–fetal interface is composed of the fetal-derived placenta and the maternal-derived decidua, whose proper establishment guarantees a successful pregnancy. For better adaption to embryo implantation, on the maternal side, endometrial stromal cells (ESCs) begin to proliferate and differentiate into large, round, cytoplasm-rich, multi-nucleated decidual cells, known as decidual stromal cells (DSCs), and this process is defined as “decidualization” in a narrow sense (12, 13). Upon embryo implantation, on the fetal side, the outermost layer of the blastocysts transforms into cytotrophoblasts (CTBs), which then generate multinucleated synctiotrophoblast (SCTs) and invasive extravillous cytotrophoblasts (EVTs) after series of chorionic villi differentiations. SCT is the outer lining of the chorionic villi that is in direct contact with maternal glandular secretions and maternal blood flowing into the intervillous space full of maternal peripheral blood, whereas EVTs get in touch with decidua and infiltrate into local lymphatics, veins, and glands, participating in placenta construction and fetal development (14, 15). Derived from the anchoring villi (the distal tips of chorionic villi in contact with the decidua), proliferative EVTs migrate into the decidua and experience two differentiation pathways toward opposite directions: The interstitial EVT invades across the decidual stroma toward the maternal spiral arteries as far as the muscular layer of the uterine wall, whereas the endovascular EVT moves in a retrograde manner down the artery to form a plug that prevents blood from entering the intervillous space and replaces endothelial cells (16). At the 10th week of gestation, when the placenta switches from histiotrophic to haemotrophic nutrition, EVT plugs dissolve, licensing abundant blood support to the fetus (17).

In the first trimester of pregnancy, decidual immune cells concede the invasion of fetal-derived cells and cooperate with the process of initial placental development and angiogenesis by forming an anti-inflammatory network and releasing pro-angiogenetic cytokines (18). In this period, up to 40% of decidual cells are leukocytes, where natural killer (NK) cells account for the most at around 70%, followed by decidual macrophages (dMφ, 20%–25%) and T cells (3%–10%), dendritic cells (DC), and B cells (18). Among these, dMφ performs crucial functions, including spiral arteries remodeling, facilitating trophoblast invasion, scavenging apoptotic cells, canonical antimicrobial function, and aiding in the construction of the immune tolerant microenvironment (19).

The tumor microenvironment (TME) is a unique milieu featuring a shortage of nutrients and scarce oxygenation, which contains tumor cells, extracellular matrix, stromal cells (such as fibroblasts, mesenchymal stromal cells, pericytes, adipocytes, blood, and lymphatic vasculatures), and immune cells (including T and B lymphocytes, DC, NK cells, monocytes, and macrophages) (20). Like the decidua, a dynamic network of tumor-related cytokines and chemokines, growth factors, and metabolic products together achieve the balance of tumor inhibition and promotion (5). As the most abundant immune population within the TME, tumor-associated macrophages (TAMs) constitute a plastic and heterogeneous cell population that accounts for up to 50% of some solid neoplasms and are associated with adverse prognoses (21). They have been demonstrated to exert multiple protumor activities that range from tumor initiation and promotion, metastasis and colonization, immune evasion, angiogenesis, and extracellular matrix remodeling (22). Importantly, because of their intrinsic plasticity, TAMs can rapidly respond to surrounding perturbations occurring in the process of tumor progression and therapeutic challenges. Moreover, TAMs prefer to enrich in a hypoxic TME milieu with poor blood supply and contribute to tumor vasculature formation by producing molecules and enzymes with proangiogenic effects (23)

**Figure 1:**
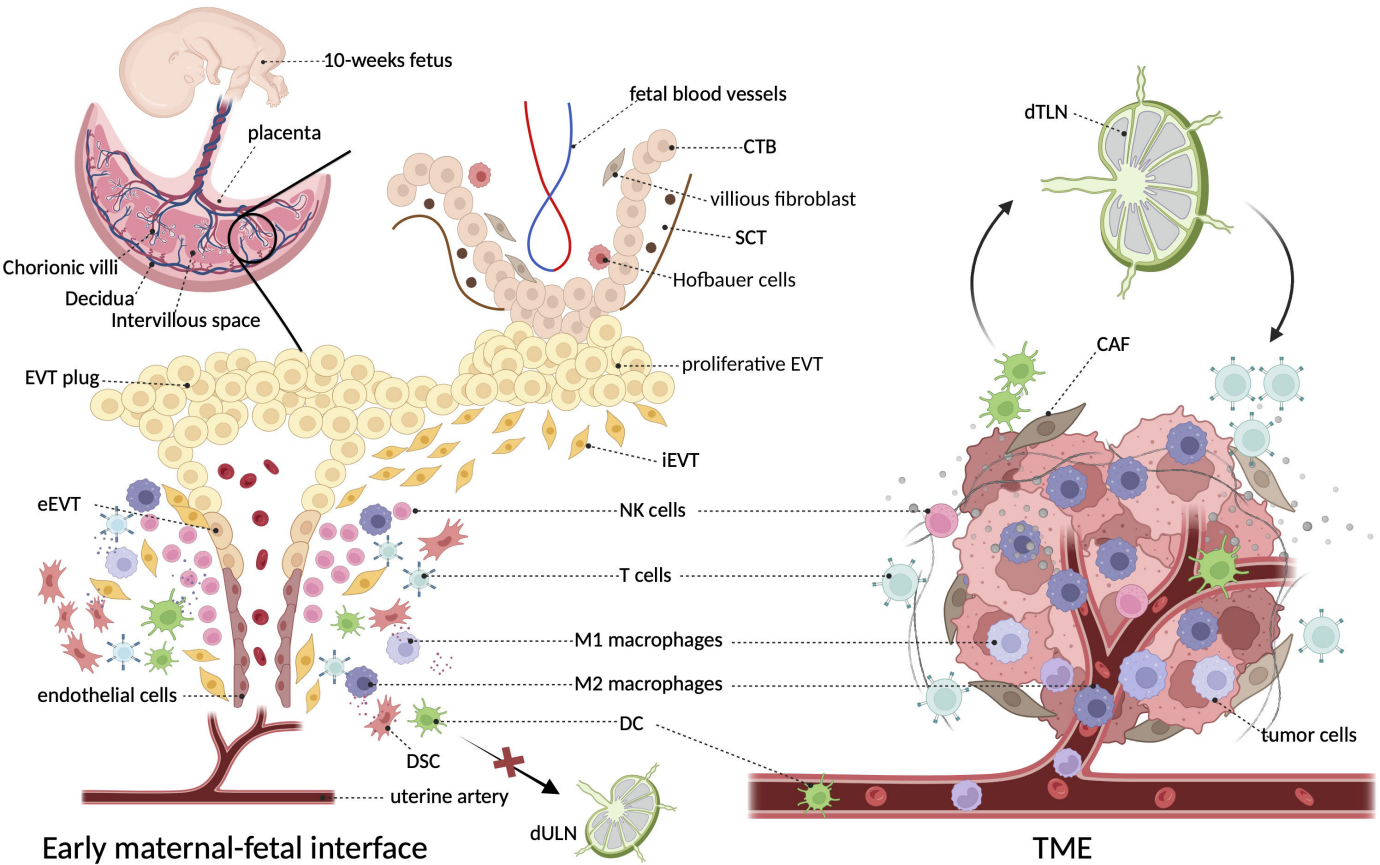
The architecture and surroundings of the immune microenvironment in tumor and decidua. The diagonal frame shows that macrophages constitute a major component of both immune microenvironments. The two microenvironments are composed of multiple cell types including tumor cells/trophoblasts, fibroblasts/stromal cells, endothelial cells, epithelial cells, and various immune cells, where macrophages constitute a major immune component (5). At the decidual face, growing invasive fetal cells come in direct contact with decidua, as well as the abundant maternal blood after the EVT plug is eroded at the beginning of the second trimester (16). Unlike the primary role of NK cells in the decidua, TME of cancer is dominantly infiltrated with macrophages (26). In particular, dendritic cells efferent from tumors can present cancer-related antigens to T cells after they enter lymph nodes, whereas decidual dendritic cells cannot migrate to draining lymph nodes due to the degeneration of lymphatic vessels (25). CTB, cytotrophoblast; CAF, cancer-associated fibroblast; DSC, decidual stromal cells; EVT, extravillous trophoblasts; SCT, synctiotrophoblast; TdLN, tumor-draining lymph nodes; UdLN, uterine-draining lymph nodes.

It can be perceived that the early maternal–fetal interface bears a notable resemblance with TME in terms of structural composition, in which tumor cells and trophoblasts act as the center surrounded by extracellular matrix (ECMs), stromal cells, and immune components, and they both have unusual vascularity. However, the proportion of various immune cells is inconsistent between the two settings, as TAMs dominate the immune environment of tumors, whereas decidual NK cells lead at the maternal–fetal interface. Different from the close relationship between tumor and tumor-draining lymph nodes (TdLNs), ESC decidualization results in a loss of lymphatics (24, 25), which restricts the DC migration and homing to uterine-draining lymph nodes (UdLNs) and avoids the susceptibility to mounting an overwhelming immune response to attack the fetus. **Figure 1** characterizes these microenvironments in both settings.

## 3 Diversity of origins and phenotypes of macrophages

**Figure 2:**
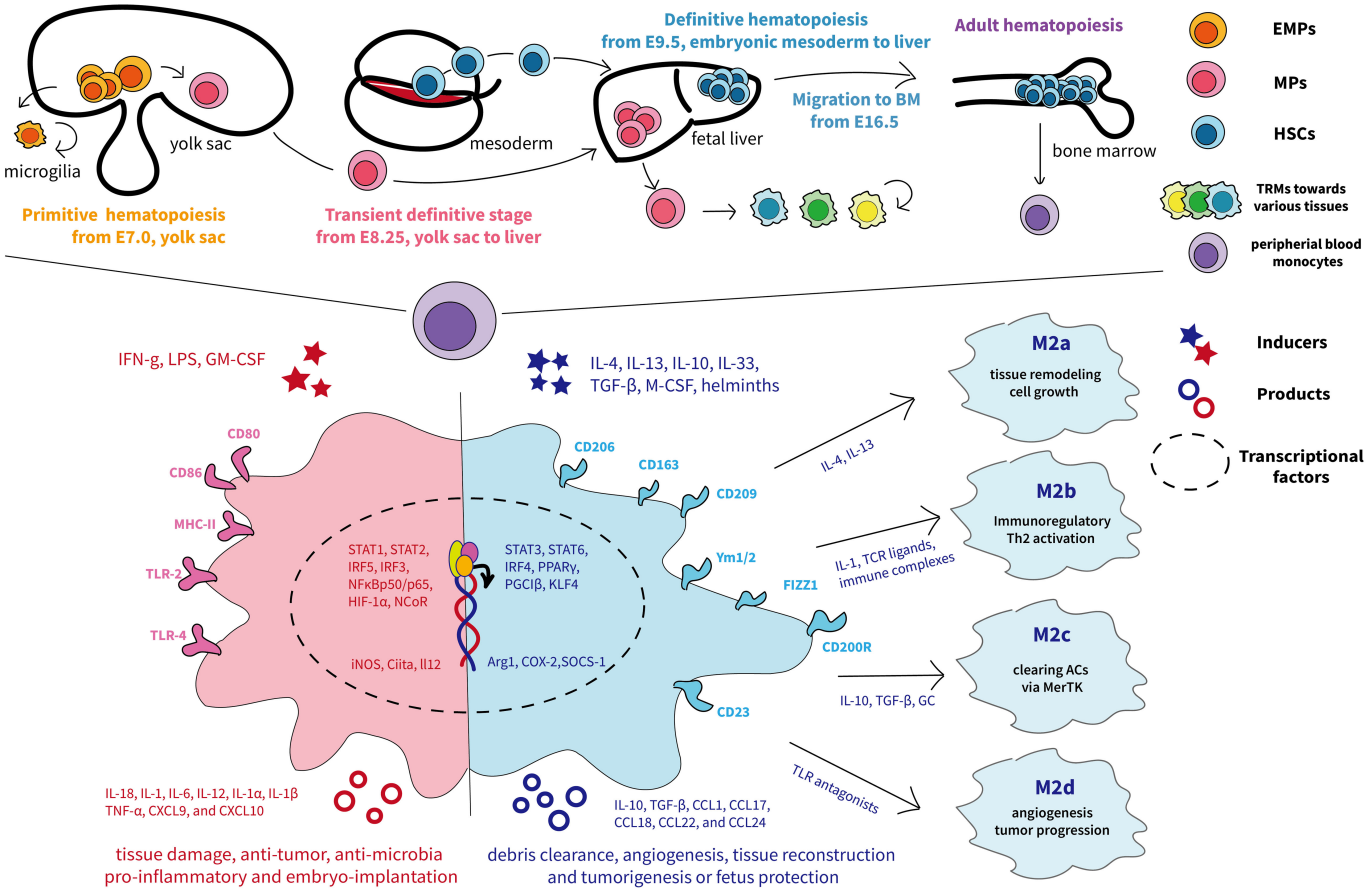
The ontogeny and phenotypic differentiation of macrophages. The top half of the graph depicts the murine fetal macrophage development. The first wave, known as primitive hematopoiesis, directly occurs in the blood islands of the extra-embryonic yolk sac at E7.5 and the yolk sac differentiates into microglia (11); the second wave is initially generated from proliferative EMPs in the yolk sac around E8.25 and subsequently arrive fetal liver, where progenitors produce fetal liver monocyte-derived macrophages, seeding diverse tissues, and differentiate into tissue-resident macrophages with renewing ability (11). The final wave relies on HSCs and begins to produce fetal hematopoietic stem and progenitor cells from approximately E9.5 in the intra-embryonic mesoderm and then transfers to the fetal liver for further maturation (30). Later, HSCs migrate to and colonize the developing fetal bone marrow by E16.5, where they remain and deliver monocytes to peripheral blood throughout adulthood (31). The different colors represent each origin of macrophages. The lower half shows the traditional M1 and M2 nomenclature, involving distinct transcription factors, phenotypes, and secretory profiles, resulting in different functions according to the complex external contexts (32). M1 macrophages are acutely stimulated by IFN-γ or LPS and secrete inflammatory factors and killing molecules against foreign invaders (26). Depending on the activating stimulus, M2 macrophages can be further divided into four different subsets consisting of M2a, M2b, M2c, and M2d (33). In particular, M2a (induced by IL-4 and IL-13) and M2b (induced by TLR or IL-1R agonists) exert immunoregulatory functions and drive Th2 responses, whereas M2c macrophages (induced by IL-10) are more related to anti-inflammatory activities against apoptotic cells and M2d macrophages (induced by TLR antagonists) are inclined to induce tumor progression (33, 34). ACs, apoptotic cells; BM, bone-marrow; CXCL, chemokine (C-X-C) ligand; CCL, chemokine (C-C motif) ligand; EMPs, erythro-myeloid progenitors; GC, glucocorticoids; GM-CSF, granulocyte-macrophage colony-stimulating factor; HSC, hematopoietic stem cells; HIF, hypoxia-inducible transcription factors; IFNg, interferon-gamma; iNOS, inducible nitric oxide synthase; MHC, major histocompatibility complex; MPs, myeloid progenitors; M-CSF, macrophages-stimulating factor; PPARγ, peroxisome proliferator–activated receptor γ; TLR, Toll-like receptor; TNF-α, tumor necrosis factor–alpha; TGF-β, transforming growth factor–beta; Ym1/2, Ym1 and Ym2.

Macrophages are the preliminary immune system to appear during embryonic development, and their exact ontogeny has been widely explored. In adults, macrophages are ubiquitous and diffuse across various bodily tissues and organs, and, for a long time, they are assumed to originate exclusively from bone marrow–derived progenitors and blood monocyte intermediates (27, 28). So far, mounting evidence has demonstrated that macrophages have additional origins in a developmental stage- and tissue-specific way (11). Fate-mapping studies and single-cell analyses in murine indicate that tissue-resident macrophages (TRMs) develop either from the macrophage’s embryonic yolk sac progenitors or fetal liver–derived erythro-myeloid progenitors (EMPs) (11). In humans, the embryo has two waves of TRMs from either yolk sac–derived primitive macrophages or yolk sac–derived myeloid-biased progenitor (YSMP)–derived embryonic liver monocytes, which corresponds to EMPs in mice (29). The diverse macrophage ontogeny is deciphered in detail in **Figure 2**.

In tumors, TAMs are essentially comprised of two origins: existing TRMs and circulating bone marrow–derived macrophages (BMDMs) (35). Monocyte-related MDSCs (M-MDSCs) are currently known as the main circulating precursor of TAMs, which are induced by a broad range of inflammatory signals to infiltrate into the tumor entity and are influenced by extreme survival conditions, like hypoxia (36). Ligand/receptors axes mediating TAMs recruitment and accumulation mainly include macrophage colony-stimulating factor/colony-stimulating factor 1 receptor (M-CSF/CSF-1R), CCL2/CCR2, CX3CL1/CX3CR1, CCL3/CCR1, CCL3/CCR5, CCL5/CCR5, and vascular endothelial growth factor–A/vascular endothelial growth factor receptor 1 (VEGF-A/VEGFR1) (37). Compared to physiological conditions, tumors are featured by a significantly higher expansion of TRMs, such as lung carcinoma, glioblastoma, and hepatocellular carcinoma (35). Evidence suggests that TAMs of different origins may coexist in tumors, and TAMs of different origins display different functions within a certain TME (35). However, recent studies suggest that different niches can direct TAMs activation profiling in a cancer type-, tissue-, and stage-specific way, highlighting a determinate role of specific TME rather than origin in the education of TAM populations (38).

Compared to TAMs, evidence covering the origin of dMφ is scarcer (30). During menstruation, an influx of macrophages derived from classical monocytes flow into secretory endometrium emerges, concomitant with the increase of macrophage-derived cytokines and proteases (31). At 18 days after fertilization, a group macrophage of fetal origin emerges in the chorionic plate, later migrating to villi at the placenta, which is referred to as Hofbauer cells (human, CD14^+^HLA-DR^−^FOLR2^+^; and mouse, CD11b^lo^F4/80^hi^CX3CR1^hi^) (39). Decidual macrophages of maternal origin (human, CD14^+^HLA−DR^hi^; mouse, CD11b^hi^F4/80^lo^), replenished from the maternal circulation by HSC-derived cells, are present in decidua basalis throughout gestation (39). These two populations account for 70% and 30% of macrophages at the maternal–fetal interface, respectively (40). CSF-1 and CX3CR1 are reported to regulate monocyte extravasation from blood vessels to the uterus (31, 41). The specific cluster of dMφ with self-renewing capacity existed since the embryonic stage of the mother and is more likely dominant to exert a homeostatic role at decidua during pregnancy than subsequent circulating monocytes (41).

Dynamic TAM phenotype drives different functions, which could influence cancer outcome. During a long time, it is commonly accepted that macrophages are classified into two distinct subsets M1 and M2 that represent two extreme activation conditions, where the former excels at fighting against pathogens to protect the host, whereas the latter is considered to be associated with immunoregulatory and pro-tumoral activities (26, 32). **Figure 2** shows the current M1/M2 nomenclature, involving distinct transcription factors, phenotypes, and secretory profiles, resulting in different functions according to the complex external contexts. However, it should be noted that M2-like macrophages should not be regarded as cancer-specific, even less TAMs (26) but rather present an alternative activated status displaying high phenotypic heterogeneity (currently thought to be four subsets) and governing functions at the interface of immunity, tissue homeostasis, and metabolism (33, 34, 42). Indeed, M2-like macrophages can occur after any immune response in healthy organisms to initiate tissue remodeling and dissolve inflammation. In cancer, macrophages crewing toward M2 status are hijacked to benefit tumor progression (43). Therefore, this binary-polarization model neglects that the adaption driven by environmental signals in the TME is flexible rather than static and could not decipher the complex activation and heterogeneity of TAMs (44). Backed by some large-scale transcriptomic studies, the model “multi-function knife” began to be widely accepted indicating that TAMs are present in a transitional intermediate state, consistent with extraordinary plasticity and a broad spectrum of activation and functional states (10), and some recently identified TAMs subsets (45–48) with specific functions have been listed in **Table 1**.

**Table 1:** The summary of the recent classification of TAMs and dMφ.

	Time	Method	Source	Subset (phenotype and function)	Marker	Reference
**TAMs**	2019	scRNA-seq	CRC, HCC, and OSCC	FCN1^+^ TAMs	FCN1, VCAN, FCGR2A, FCGR3A, S100A6, S100A8, S100A9, and S100A12	(45–47)
	2017	scRNA-seq	CRC, HCC, and NSCLC	C1Q^+^ TAMs	C1QA/B/C, APOE, TREM2, and SLC40A1	(46–48)
	2020	scRNA-seq	CRC	SPP1^+^ TAMs	SPP1, IL1RN, OLR1, VEGFA, EREG, FN1, C15ORF48, PHLDA1, AQP9, TNS3, and NDRG1	(46)
**dMφ**	2011	Flow cytometry	NP decidua of 6–12 weeks	CD14^+^CD11c^hi^: lipid metabolism and inflammation, process protein Ag CD14^+^CD11c^lo^: extracellular matrix formation, muscle regulation, and tissue growth	CD14, CD45, and CD11c	(49)
	2011	Flow cytometry	NP decidua of 7–11 weeks	CD163^high^ CD206^high^ CD209^high^ NRP-1^high^ ICAM-3^low^	CD14, CD163, CD206, CD209, and NRP-1	(50)
	2018	scRNA-seq	NP decidua and placenta of 6–14 weeks	dM1: remodeling dM2: self-renewal and remodeling	ITGAX and IL-10	(51)
	2018	Flow cytometry	NP decidua of 6–8weeks	CCR2^+^CD11c^hi^: pro-inflammatory characteristics CCR2^−^CD11c^hi^: anti-oxidative and anti-inflammatory CCR2^−^CD11c^lo^: antigen presentation	CCR2, HMOX1, MARCO, MHCII, and IL1B	(49, 52)
	2021	scRNA-seq and ATAC-seq	Decidua of 15 NP (7–24 weeks) and nine RPL (8–50 weeks) women	CD11b^+^: M1 polarization characteristics CD11c^+^: M2 macrophage homeostasis in the decidua	CD11b^+^: S100A10, S100A6, S100B, and S100A4; CD11c^+^: PLAU, DAB2, EGR1, SEPP1, and MAF	(9)
	2021	scRNA-seq	Decidua of six NP (7 weeks) and six RPL (5 weeks) women	Ten clusters according to transcriptional states, suggesting that dMs do not fit the conventional M1/M2 classification	IL1BIL6, IL10, MS4A7, CCL2, CCL3, and CCL4	(53)
	2021	scRNA-seq	Decidua of 10 NP and 14 RPL women (6–8 weeks)	CD14-expressing dM cells	CD14, ITGAX, CXCL8, CXCL3, C1QA, C1QB, and C1QC	(54)

CRC, colorectal cancer; dM, decidual macrophages; HCC, hepatocellular cancer; NSCLC, non–small-cell lung cancer; NP, normal pregnancy; OSCC, oral squamous cell carcinoma; RPL, recurrent pregnancy loss; scRNA-seq, single-cell RNA sequencing. ATAC-seq, Assay for Transposase Accessible Chromatin with high-throughput sequencing.

dMφ possesses functional and quantitative plasticity with remarkable adaptation to changing environments (55). Initially, seminal plasma triggers an essential inflammatory milieu that contains an increase in M1-skewed macrophages in the peri-implantation uterus (56). Upon EVT attachment onto the endometrial lining and invasion into decidua, it transits to a mixed profile of M1/M2 macrophages (14). A predominantly M2 phenotype gradually forms along with uterine vascular remodeling, culminating with completed placental development at the beginning of the second trimester (57). These M2-like dMφ promote immune tolerance to semi-allogenic fetuses and protect fetal growth till parturition, which is a major source of interleukin-10 (IL-10) at decidua. Finally, the surge of M1 dMφ at parturition, together with an outflux of inflammatory cytokines, suggests their important role in triggering the term delivery (58). DMφs exhibit excellent plasticity and are sensitive to small changes in the microenvironment, including nutritional status, smoking habits, inflammatory conditions, and physiological stress. Inappropriate macrophage plasticity, whenever it occurs, could impart adverse effects on pregnancy outcomes, leading to complications like infertility, recurrent pregnancy loss (RPL; defined as loss of two or more consecutive pregnancies), preeclampsia [PE; defined as a pregnancy-related syndrome with high blood pressure (≥140/90 mmHg) and/or proteinuria (≥ 0.3 g/L)], fetal growth restriction (FGR; defined as a failure of fetus to reach its expected biological growth), and preterm delivery (59). In the past decades, scientists have made efforts to identify dMφ subgroups and confirm phenotypes of dMφs related to the maintenance of healthy pregnancy (49–54, 60), which is summarized in **Table 1**.

## 4 Factors regulating macrophage phenotype and function

### 4.1 Metabolic reprogramming

#### 4.1.1 Hypoxia

With restricted oxygen availability, tumor cells undergo metabolic adaptations and bear the capacity to initiate high rates of aerobic glycolysis, which generates a nutrient-deficient TME (61). In this context, TAM states and phenotypes can be adjusted by disparities in tumor tissue oxygenation, which benefits tumor cells to overcome nutritive deprivation and convert the TME into more hospitable sites (2). More specifically, M2 cells tend to be in hypoxic, necrotic, or perivascular tumor sites where they dampen anti-tumor inflammation, whereas M1 cells enrich normoxic regions (62). Although hypoxia generates both pro-inflammatory markers, like tumor necrosis factor–alpha (TNF-α), IL-1β, and inducible nitric oxide synthase (iNOS), as well as anti-inflammatory markers such as IL-10 and arginase 1 (ARG1), TAMs shift mainly toward the M2-like phenotype (63). Beyond enhancing TAMs homing to tumor sites via hypoxia-induced release of chemoattractant, hypoxia also fine-tunes the expression level of major histocompatibility complex II (MHC-II) on TAMs to induce “immune invisibility” of tumor-specific antigen, as MHC-II^lo^ TAMs always populate hypoxic areas, whereas MHC-II^hi^ TAMs prefer normoxic (64).

Compared to TME located in hypoxic niches (0.1%–5% O_2_) of solid tumors (64), the hypoxic level of the whole placenta is more moderate. Although the dramatic changes of O_2_ levels in villous regions during the first (2%–3%) and second trimester (8%–10%) as the perfusion of the placenta is thoroughly established, O_2_ levels at decidua (5%–6%) maintain relatively constant during early gestation, which is comparable to the unpregnant uterus (39, 65). The sites located near the uterus arteries even bathe within a rich oxygen supply (up to 10%–14% O_2_) (39). Thus, hypoxia seems to make less impact on dMφ phenotypic transformation compared to TAMs. Still and all, the proper extent of hypoxia is indispensable to a thriving placenta (5). In the early stage, hypoxia aids peripheral NK cells in homing to the uterus and promotes normal expansion and development of Hofbauer cell progenitors (66). In pregnancy complications where misfunctioned dMφ can be seen, like PE and RPL, insufficient uterine spiral arteries remodeling and uteroplacental ischemia cause extreme hypoxia (67), suggesting the importance of a fine balance of oxygen concentration on dMφ differentiation and function.

#### 4.1.2 Lactic acid

Lactic acid (LA) is a metabolic product produced from glucose through glycolysis and the conversion of pyruvate by lactate dehydrogenase (LDH) under oxygen-deprivation conditions (68). In extremely hypoxia TME, the glucose restriction and low pH mediated by tumor-derived LA induce a poorly glycolytic M2-like profile along with the high levels of VEGF and ARG1, fatty acid oxidation (FAO) upregulation, and weak capacity for antigen presentation (69). It has also been reported that modulation of LA levels can redistribute M2-TAM subsets and upregulate PD-L1 to assist tumor immune escape via inducing T-cell apoptosis (70).

At the maternal–fetal interface, trophoblasts and DSCs are a major source of LA, especially during peri-implantation and decidualization (68). Unlike tumors, decidua-derived LA exerts a dual role in regulating macrophage polarization according to the concentration of oxygen and LA (71). Under normoxia, light trophoblast-derived LA promotes macrophage polarization to the M2 phenotype by upregulating mitochondrial oxidative phosphorylation (OXPHOS) and VEGF expression independent of hypoxia-inducible transcription factor–1α (HIF-1α), whereas, upon hypoxia, vast trophoblast-derived LA promotes M1 phenotype polarization by upregulating glycolysis and iNOS mediated by HIF-1α/SRC/LDHA pathway (71). Clinically, the maternal–fetal microenvironment in patients with RPL is characterized by enhanced LA accumulation and exaggerated hypoxia, suggesting that glucose metabolism may be a therapeutic target by reining dMφ phenotype and function (72).

#### 4.1.3 Amino acid metabolism

Macrophages from both human and mouse TME are desperate for non-essential amino acids, thus depleting the local environment of amino acids necessary for optimal effector function of immune cells and thus subverting immunosurveillance (73). Among these, arginine metabolism is a key regulator (73). The hypoxic state of the tumor prefers the catabolism of Arg1 over iNOS, resulting in more M2-like polarization and elevated tumor-supporting factors into TME (74).

In addition, the concentration of tryptophan (Trp) and its by-product Kynurenine (Kyn) in the local microenvironment also contribute to the polarization of macrophages (75). Trp-consuming enzyme Indoleamine 2,3-dioxygenase (IDO) is highly expressed on TAMs and dMφ and causes a functional loss on NK and T cells (43, 44). In addition to the deprival of Trp, immunosuppressive Kyn interacts with the ligand-activated transcription factor aryl hydrocarbon receptor (AHR) and drives the generation of Tregs and tolerogenic myeloid cells and the upregulation of Anti-programmed cell death 1 (PD-1) in CD8^+^ T cells, modulating M2-like macrophages activity (76). The blockade of IDO could reduce the recruitment and phagocytosis of macrophages, switching them to M1-like phenotype (76). Clinically, a remarkable decrease of IDO^+^ dMφ is observed in patients with RPL; meanwhile, the treatment with IDO inhibitor, 1-methyl-D-tryptophan, during early pregnancy leads to obvious uterus inflammation and fetus loss (77).

#### 4.1.4 Other metabolites

As recently reported, lysophosphatidic acid (LPA), an important intermediate in lipid metabolism produced by uterine epithelium, binds nuclear receptor peroxisome proliferator-activated receptor gamma (PPARγ) within dMφ and thus induces the transcription of M2-differentiation genes and facilitates the accumulation of decidua-resident macrophages, which favors trophoblast invasion and placental development (65). In line with, clinical observation found that a low expression of LPA receptor in villous decidua is associated with the risk of early RPL. In addition to this, the effect of LPA on TAM formation and polarization also has been reported in ovarian carcinoma (78).

In addition, because of the unique maternal–fetal interface components, like trophoblasts and DSCs, and pregnancy-related hormones, there may be some distinct mechanisms driving dMφ toward tolerant phenotypes that TMEs do not involve. For example, fructose-1,6-bisphosphate (FBP), a crucial glucose metabolite during decidualization, is found to increase the secretion of IL-27 from DSCs, which induces M2-like dMφ differentiation and suppresses their production of pro-inflammation molecules (79). Given the low level of FBP observed in decidua and plasma of patients with RPL (79), FBP supplementation could be a promising strategy to prevent fetal loss by targeting IL-27–triggered M2-like macrophages.

### 4.2 Epigenetic regulation

Via modifying the differentiation and functional programming of macrophages, epigenetic control provides a highly flexible mechanism for the activation and deactivation of transcription in macrophages in response to diverse stimuli in the TME, acting as a basis of plasticity. Firstly, DNA methylation. There are pieces of evidence that DNA methyltransferases (DNMTs) have a specific effect on the formation of macrophage phenotypes. Ubiquitin-like protein containing plant homeodomain (PHD) and RING finger domain 1 (UHRF1) maintains the DNA methylation status by recruiting DNMT1 (80). A recent study found that decreased UHRF1 in trophoblasts promotes the expression of pro-inflammatory IL-1β, which recruits monocytes to decidua and shifts them into the M1 phenotype, further exacerbating RPL (80). In cancer, DNA methylation is critical for the suppression of the expression of tumor suppressor genes, whereas the loss of DNA methylation mediated by removal of methyl groups leads to the overexpression of oncogenes. For example, the expression of demethylase ten-eleven translocation 2 (TET2) is increased in TAMs in the setting of melanoma in an IL-1R–MyD88 pathway–dependent way (81).

Secondly, histone methylation and acetylation also participate in the epigenetic regulation of macrophages. In tumors, histone deacetylase (HDAC) class I (HDAC3) is central in M2 activation, whereas HDAC class II (HDAC 4, 5, 6, and 7) mainly targets the M1 subgroup (44). Enhancer of zeste homolog 2 (EZH2) is the enzymatic subunit of polycomb repressive complex 2 (PRC2), which can catalyze trimethylates lysine 27 of histone H3 (H3K27me3) to mediate gene transcriptional silencing and chromatin compaction (82). It has been reported that the suppression of EZH2 in various malignant tumors like colorectal cancer (CRC) shifts TAMs toward the M1 phenotype via decreasing the H3K27me3 levels on the promoters of STAT3 (83), which is beneficial for tumor therapy, whereas a significantly decreased expression of EZH2 was found to be associated with adverse pregnancy outcomes such as RPL (84). Expectedly, inhibition of EZH2 in trophoblasts disturbed the phenotypic differentiation of macrophages, transforming M2-like into M1‐like dMφ, via reducing the production of transforming growth factor–beta (TGF‐β), IL‐10, IL‐6, IL‐4, CXCL‐16, and PD‐L1 (84). Moreover, decreased HDAC8 expression downregulates the level of M2 marker genes like CD163 in dMφs, which may disturb immune tolerance and induce pro-inflammatory response at the maternal–fetal interface and be relevant to RPL (85).

Moreover, noncoding RNAs are another modification to macrophages. Abundant evidence shows more than 100 micro-RNAs (miRNAs) impinge on macrophage polarization and function, forming a complex post-transcriptional regulating network (86). In tumors, for example, miRNA regulates TAM polarization to the M1 phenotype, via miR-155, miR-181, and miR-451, or to the M2 phenotype, via miR-99a, miR-146a, miR-125a, and miR-145-5p (73). Regarding dMφ, miR-19a-3p and miR-130b-3p, and miR-10a-5p and miR-150–5p have been reported to inhibit the M1-marker gene expression and increase the production of M2-phenotype markers. Like TAMs, miR-155 derived from dMφ also promotes M1 polarization while impairing M2 status (4), the alteration of which is associated with gestational complications such as miscarriage and PE.

### 4.3 Environmental growth factors, cytokines, and chemokines

The role of secreted or surface molecules in cancer progression and TAM phenotypic transformations remains inadequate, which can be explained by TAMs’ plasticity shaped by the heterogeneity of source of certain molecules, tumor types, and stages, and systemic immunity of hosts. A certain molecule could exert diverse functions in regulating macrophage polarization according to specific microenvironments or subgroups of macrophages, such as Dectin 1 (87, 88) and CCL2/MCP-1 axis (89). Given various essential modulators involved in macrophage polarization in tumors and pregnancy, targeting can help switch or regulate macrophage polarization, thus probably facilitating potential immunotherapy. **Table 2** summarizes the effect of current reported environmental factors on the polarization of macrophages from tumors and decidua (90–94, 96–106).

**Table 2:** The various environmental factors that affect macrophage polarization and phenotype.

Interference factor	Source	Influence on macrophage phenotype	Clinical relation	Reference
GM-CSF	Trophoblast and DSCs	M1 polarization	Tumor; pregnancy	(35, 90)
M-CSF	Trophoblast and DSCs	M2 polarization	Tumor; pregnancy	(35, 90)
VEGF	Trophoblast and DSCs	M2 polarization	Pregnancy	(91)
CCL2/MCP-1	DSCs; tumor cells	Balanced M1/M2 dMφ	LLC; pregnancy	(89, 92)
TGF-β	CA-MSCs	Immunosuppressive M2-like phenotype	Cancer	(93)
CX3CL1	CA-MSCs	Immunosuppressive M2-like phenotype	Cancer	(89, 93)
IL-10, IL-4, and IL-13	Trophoblast	M2-like subtype dMφ	Pregnancy	(50)
GAS6	DSCs; apoptotic cells	Immunosuppressive phenotype	RSA; cancer	(94, 95)
GdA	DSCs	dMφ-like phenotypes	Pregnancy	(96)
IL-33	Dying cell; macrophages	M2 polarization	RSA	(97)
IL-34	Trophoblast; DSC	Immunoregulatory phenotype	Pregnancy	(98)
CXCL16	Trophoblast	M2 polarization; lower IL-15 level	Pregnancy	(99)
IL-6	Tumor cells; trophoblast	M2 phenotype	TNBC; pregnancy	(100, 101)
Hyaluronan	Trophoblast	M2 phenotype	Pregnancy	(102)
RANKL/RANK	DSCs	M2 phenotype; higher adhesion molecules	Pregnancy	(103)
Progesterone	Ovary, placenta, and fetus	M2-like phenotype	Pregnancy	(104)
HCG	Placenta	M2 polarization	Pregnancy	(105)
PGE2	DSCs	M1 polarization	RSA	(106)
Dectin 1	Trophoblast	M2 polarization	GBM; PDAC	(87, 88)

CA-MSC, cancer-associated mesenchymal stem cells; DSC, decidual stromal cell; GM-CSF, granulocyte-macrophage colony-stimulating factor; GdA, glycodelin-A; GBM, glioblastoma; HCG, human chorionic gonadotropin; LLC, Lewis lung cancer; PGE2, prostaglandin E2; PDAC, pancreatic cancer; RSA, recurrent spontaneous abortion; TNBC, triple-negative breast cancer.

## 5 Macrophages function in immune tolerance

### 5.1 TAMs in tumor immune escape

#### 5.1.1 TAMs interact with stromal cells

As the most abundant stromal cells in the TME, cancer-associated fibroblasts (CAFs) promote the recruitment of monocytes and their transformation into M2 phenotype via secreting multiple regulatory molecules, such as IL-6, M-CSF, monocyte chemoattractant protein-1 (MCP-1, or CCL2), and stromal cell–derived factor 1 (SDF-1, or CXCL12) (107). More importantly, CAFs can motivate the immunosuppressive properties of TAMs, including higher PD-1 expression (45). Reciprocally, M2-like TAMs regulate CAF activation and progression and promote the EMT process, which consequently creates a positive loop between CAFs and TAMs, together promoting tumor evasion and survival (107).

#### 5.1.2 TAMs regulate the function of other immune cells

TAMs can directly inhibit cytotoxic immune cell responses. Firstly, TAMs affect the phenotypic and functional differentiation of T cells via reversing the anti-tumor cytotoxic T lymphocyte (CTL) and CD4^+^ Th1 cells to tolerant Th2 cells, Th17 cells, and Tregs cells in a manner dependent on inhibitory cytokines, such as TGF-β and IL-10 (36). TAMs are also involved in tumor immune microenvironment (TIME) formation through their expression of immune checkpoints such as PD-L1/L2 and CD80/86 that bind to their receptors (PD-1/cytotoxic T- lymphocyte-associated protein 4 (CTLA-4)) that constitutively express on T cells, directly restraining the function of activated anti-tumor T cells (36). Subsequently, Th cells, in turn, remodel tumor TME partly by altering the polarization direction of macrophage, as Th2 cells secrete IL-4, IL-5, and IL-10 to induce more M2 macrophages (44). In addition, TAMs play a crucial intermediary role through both soluble mediator crosstalk and cell-to-cell contact to weaken tumor-associated NK cells. TAMs harboring inhibitory molecules like human leukocyte antigen-G (HLA-G) and human leukocyte antigen-E (HLA-E) interact with NK/T cells via coinhibitory signal molecules ILT2 and NKG2, respectively (108), which have been proved to neighbor TAMs physically *in vivo* and appear “disarmed.” In addition to the direct contact, the secretion of IL-10 and TGF-β by TAMs also educates NK cells toward an immunosuppressive state (109). As previously mentioned, TAM-mediated metabolic activities, including the depletion of metabolites (such as L-arginine and Trp) also promote Treg activity and impair both CTL proliferation and interferon-gamma (IFN-γ) production, thus engaging in the immune-suppressive TME (61). Moreover, IL-10 released by TAMs suppresses IL-12 expression by conventional DCs in tumors, resulting in a diminished CTL response (36).

TAMs also recruit immunosuppressive populations and build positive feedback loops to facilitate the expansion of each other and reinforce the suppressive TIME. TAMs have been shown to promote Treg cell recruitment to human carcinomas via CCL22 and CCL1, which are the respective ligands of CCR8 and CCR4 on Treg cells (23). In the stage of tumor progression, TAMs also secrete high levels of IDO1 and CXCL10 to attract surrounding Treg cells (36). In turn, Treg cells from TME provoke the production of IL-10, TGF-β, and CD73 by TAMs and increase expression of negative co-inhibitory molecules such as PD-L1 in an autocrine way, which later binds to effector T cells and restrains their anti-tumor activity (110).

TAMs also interact with unconventional T cells in tumor immunity, although not all lead to immune suppression. Invariant natural killer T cells (iNKT) were found to directly suppress M2-like TAMs and promote the survival of M1-like macrophages in a CD40/Fas-dependent way, thereby weakening prostate cancers in a transgenic mouse model (111). Moreover, TCRαβ^+^CD4^−^CD8^−^NK1.1^−^ innate αβ T cells (iαβT) promote M1-like proinflammatory reprogramming of macrophages in a CCR5-dependent manner with enhanced antigen presentation and T-cell chemoattraction, this way improving CD4^+^ and CD8^+^ cytotoxic T-cell toward the tumor (112). As mentioned above, γδ-T cells can be stimulated by CCR2^+^TAMs to secrete IL-17, thus orchestrating a pro-metastatic response (113).

#### 5.1.3 TAMs interact with tumor cell

It prevails that malignant tumor cells are encouraged to evade immune surveillance and “cool” the immune response (114). In this case, it is another key underlying mechanism for tumors avoiding immune surveillance that TAMs being hijacked and manipulated by tumor cells prefer to establish a tolerant TME. Initially, tumor cells highly express self-labels like CD47, β2M, and CD24, which can phosphorylate ITIMs and thus downregulate the phagocytic function of macrophages (63). Furthermore, tumor cells produce a plethora of cytokines and chemokines to bind to receptors on the surface of TAMs, handling their expression of relevant immunosuppressive genes. Manipulated TAMs thus gain secretion of various tumor-promoting factors including CCL18, IL-10, and TGF-β while inhibiting the release of cytotoxic factors like IL-2, IL-12, TNF-α, and IFN-γ, impeding the anti-tumor capacity of other immune components and prolong tumor survival (115).

#### 5.1.4 Immune checkpoint molecules involved in TAMs

TAM is a major source of immune checkpoint molecules in the TME. In addition to the PD-1/PD-L1 and CTLA-4/CD86, TAMs can also induce immune escape along with other immune checkpoints, such as IDO/AHR, T-cell immunoglobulin and mucin domain protein 3 (Tim-3)/Galectin-9, and Fas/Fas-L (44). A recent study has found that Phosphatase and tensin homolog (PTEN)-deficient glioblastoma (GBM) cells secrete high levels of Galectin-9 via the AKT-GSK3β-IRF1 pathway, which drives M2 polarization by activating Tim-3 (116). Su et al. have revealed an unexpected immunosuppressive effect of macrophages that undergo antibody-dependent cellular phagocytosis by overexpressing PD-L1 and IDO, via inhibiting both NK-cell– and T-cell–mediated cytotoxicity (117). Clinically, the PD-1 expression on TAM of human cancers elevates over time with the stage of the disease, which downregulates TAM proliferation and activation, accelerating tumor cells’ escape (118).

### 5.2 DMφ in immune tolerance at the maternal–fetal interface

#### 5.2.1 Crosstalk between dMφ and decidua stroma

DSCs, the largest population at the decidua, are critical in maintaining a successful pregnancy not only by the production of hormones such as prolactin but also by shaping the local immunological microenvironment via cytokines and chemokines (30). Initially, DSCs promote the survival of CD14^+^ blood-derived monocytes and induce them into the CD14^bright^CD163^+^CD209^+^CD86^dim^ phenotype and trigger the expansion and phenotypic shift of dMφ mainly via M-CSF and TGF-β signaling (119). Subsequently, chemokines such as CCL2, secreted by DSCs, are a key regulator to maintain the fine M1/M2 mixed immune status (120). Growth arrest–specific factor 6 (GAS6) produced by DSCs also favors polarizing dMφ toward an M2-like phenotype and induces cell proliferation (94). Compared with normal pregnancy, less GAS6 secretion by DSCs in early miscarriage suggests a critical role of dialog between DSCs and dMφ in maintaining a healthy pregnancy (94). Similarly, TAM receptors (Tyro3, Axl, and MerTK), the cognate receptors of GAS6 on apoptotic cells, are also expressed on TAMs and skew polarization toward a pro-tumor M2-like phenotype, participating in immune homeostasis and tumor progression (95).

#### 5.2.2 Crosstalk between dMφ and trophoblast

Analogous to tumor cells, trophoblasts secrete high levels of multiple signaling molecules to recruit and educate immune cells and establish a tolerant environment at the maternal–fetal interface (121). In general, trophoblast-derived factors, including IDO, TGF-β, IL-10, M-CSF, IL-34, IL-6, CXCL16, and PD-L1, play critical roles in shaping dMφ into a fetus-friendly immune population (122, 123). The PD-1/PD-L1 axis modulates dMφ polarization to a phenotype subset that modulates the maternal–fetal tolerance, which inhibition has been proven to associate with increased fetal loss (124). Intrinsically, trophoblasts tend to upregulate nonclassical HLA class Ib molecules HLA-E, HLA-F, and HLA-G, to provide inhibitory signals to a broad of immune cells, including NK cells, T cells, and myeloid cells via surface inhibitory receptors CD94/NKG2A, KIR3DL1, immunoglobulin-like transcript 2 and 4 (ILT-2/4), and KIR2DL4 (125), among which, ILT-2/4 is especially enriched on macrophages. Lee et al. found that sHLA-G5 polarized macrophages toward the M2 phenotype, with higher phagocytic activity and increased expression of IDO, IL-6, and CXCL1, which impaired the proliferation and cytotoxic function of T cells and promoting trophoblast invasion (126). During normal pregnancy, the plasma level of sHLA-G5 increases in the first trimester and gradually declines as the pregnancy advances, and the low sHLA-G level in early gestation often relates to pregnancy complications, such as RPL, PE, and FGR (127).

In addition, trophoblast debris has been reported to modulate the expression of cytokines in macrophages, upregulating the expression of inhibitory molecules, like PD-L1, IDO, IL-1Ra, IL-6, and IL-10, while reducing the expression of costimulatory molecules, such as MHC-II molecules and intercellular adhesion molecule 1(ICAM-1) and pro-inflammatory cytokines including IL-8 and IL-1β (14). In turn, Mφ-derived cytokine IL-33 acts as a critical factor for placental growth in early pregnancy, inducing the proliferation of trophoblasts (59).

#### 5.2.3 Crosstalk between dMφ and other immune cells

In the first trimester of pregnancy, dMφ exercises a strong influence on the regulation of immune tolerance in the maternal–fetal interface (18). As the dominant APC, dMφ plays a role in regulating T-cell activation (127). Two decades ago, the suppressive effect of dMφ on the proliferation of effective T cells was first described in early pregnancy (128). DMφ also attracts and promotes the proliferation of Treg cells, which are in the control of maternal immune effector functions and in preventing fetal rejection (30).

Various immune checkpoints prevent an overreacted maternal inflammatory response to fetal antigens during pregnancy. Compared with peripheral blood monocytes, decidual macrophages suppress the production of Th1-type cytokine IFN-γ by decidual T cells via PD-L1/PD-1 interactions (59). Tim-3+ dMφ subset induces the exhaustion or apoptosis of cytotoxic T lymphocytes and favors Th2 and Treg bias in decidual CD4+T cells (129), and Tim-3 blockade has been proven to cause more fetal loss (90). Given pre-clinical cancer studies that found co-blockade of the PD-1/Tim-3 pathway endowed successful control over tumor growth, whether the joint effect of Tim-3 and PD-1 also make optimal protection to the fetus is attractive. Indeed, compared with the single intervention, the combination of PD-L1and Gal-9 fusion proteins are proven to exhibit obvious advantages in lowering the risk of miscarriage, trophoblast invasion, and fetal vascular development, indicating a synergistic effect of co-inhibitory pathways to better pregnancy outcomes (130).

As the most abundant leukocytes during pregnancy, dNK cells reside in close contact with decidual CD14^+^ myelomonocytic cells, indicating a potential crosstalk between them. Indeed, TGF-β secreted from dMφ restrains dNK killing toward fetal cells (131). Moreover, CXCL16 from trophoblasts decreases IL-15 secretion in dMφ (99). As an important cytokine regulating differentiation, maturation, and survival of NK cells, the reduction of IL-15 impairs NK cytotoxicity to establish immune tolerance (99). Following interaction with dNK cells, dMφ expresses IDO, which, in turn, induces the expansion of Tregs through TGF-β production or CTLA-4–mediated interactions (132).

#### 5.2.4 Intrinsic phagocytosis of dMφ helps inflammation prevention

Anyway, it cannot be ignored that as a type of professional APC the intrinsic capacity of macrophages is phagocytosis. Foreign pathogens and shedding trophoblast debris accumulated at the maternal–fetal interface become an important source of provoking inflammation and aggregating cytotoxic immune cells, which is likely to disturb the fetus-protecting homeostasis (127). In this case, either pathogen-associated molecular patterns of pathogens or damage-associated molecular patterns from apoptotic cells could help recruit decidual macrophages to the site of inflammation to prevent harmful inflammatory stress (133). Meanwhile, the pattern recognition receptors on the surface reverse these macrophages to an M1-like phenotype temporally, suggesting that they retain the role of canonical antimicrobial (127). Interestingly, how the manipulated dMφ by foreign agents make an effect on pregnancy outcomes, pro-M1 or pro-M2, is specifically related to distinct virulent strains of intrauterine pathogens, suggesting the complicated plasticity of dMφ answering external stimuli (134).

**Figure 3:**
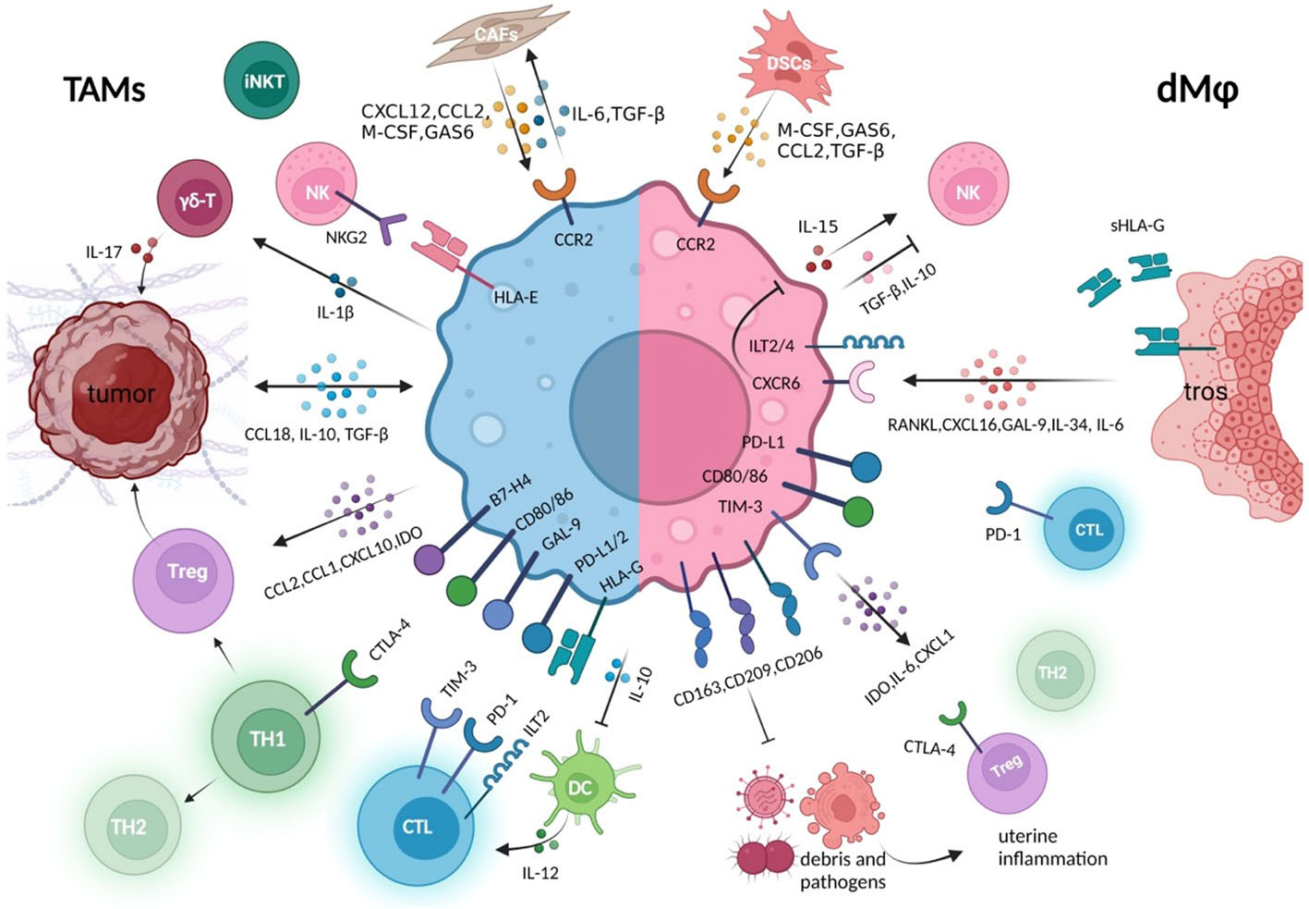
Crosstalk between macrophages and other immune components in the tumor immune microenvironment or at the maternal–fetal interface. Whether in the tumor or decidual immune microenvironment, macrophages establish close connections with other cell components to jointly create an environment of immune tolerance, which is conducive to the survival of rapidly expanding tumor cells and trophoblasts, respectively (5, 8). These macrophages either express high level of co-inhibitory molecules that suppress cytotoxicity of CTL, promote the generation of Tregs, and promote the conversion of Th1 to Th2 type, either produces various immune regulators, to inhibit the killing capacity of NK and effector T cells (36, 67). In the tumor microenvironment, macrophages maintain closer hormonal communication with tumor cells and CAFs, forming positive feedback, which also exists between DSCs and decidual macrophage crosstalk (107, 119). During the rapid development of the fetus and its belongings, the rapid clearance of trophoblast debris by decidual macrophages also prevents potential inflammatory response. CAFs, cancer-associated fibroblast; DSCs, decidual stromal cells; tros, trophoblast; CTL, cytotoxic T cells; GAS6, growth arrest–specific factor 6.

Above all, in the settings of tumor and pregnancy, macrophages, at the core of immunosuppressive cells and cytokines networks, are educated by tumor cells or trophoblasts to assist tumor immune evasion or fetus protection through their capacity of recruiting multiple immune components containing cells, cytokines, and inhibitory immune checkpoints. Meanwhile, dMφs also contribute to the defense against potential intrauterine infections, acting to suppress inflammatory stress that may disturb normal pregnancy. The similarities and distinctions of how TAMs and dMφs create an immunosuppressive environment are described in **Figure 3**.

## 6 Macrophages in angiogenesis

### 6.1 TAMs exert a crucial effect on tumor neo-angiogenesis

Like the placenta, a tumor as a developing organ requires blood vessels to transport oxygen and nutrients, which can exponentially accelerate the tumor’s growth. The onset of neovascularization is orchestrated by various mechanisms, among which TAMs play a leading role in angiogenesis by promoting endothelial cell proliferation, migration, and survival, as well as ECM remodeling (135). During this process, hypoxia and LA regulate the effects of TAMs on the tumor-associated blood vessels via finely tuning the activation of TAMs and stimulating pro-angiogenic gene transcription in a HIF-α–dependent manner, such as *VEGFA*, *TIE2*, and neuropilin-1 (*NRP1*) (62, 136, 137). Endothelial cells can also exert an effect on TAMs to maintain their proangiogenic phenotype metabolically. Among these, PPAR-γ is suggested to take part in the interaction between macrophage polarization and tumor angiogenesis (138), on which endothelial cells rely to promote the alternative activation of TAMs (139), and induces the transcriptional accumulation of HIF-2α (140).

Moreover, M2-like TAMs induced by tolerant TIME promote tumor angiogenesis through the secretion of pro-angiogenic factors, including VEGFA, FGFs, CXCL8, CXCL12, WNT7B, and BV8, pro-inflammatory cytokines, namely, IL-1β, IL-6, and TNF, as well as pro-angiogenic proteases, including matrix metalloproteinases (MMPs) and cathepsins (141). Among those, VEGFA guarantees both angiogenic switch and abnormal vasculature featured by vascular permeability, thereby favoring tumor intravasation and distant metastasis (142). MMPs mobilize ECM-bound VEGFA and enable their binding to VEGF receptor 2, which is expressed on endothelial cells, triggering angiogenesis (143).

The angiopoietins (Ang-1 to Ang-4) are a family of angiogenic growth factors primarily involved in vessel stabilization in the latter stages of angiogenesis, which signal through membrane-bound tyrosine kinase receptors (Tie-1 and Tie-2). Ang-2 as one of the best-characterized Ang family numbers has also been shown to play a role in the recruitment and education of TAMs (144). Hypoxic TME induces the expression of CXCL12 and Ang-2, which stimulates the recruitment and perivascular accumulation of CXCR4^+^TIE2^+^ TAMs (145). Subsequently, the paracrine interaction between TAMs and endothelial cells that secrete Ang-2 facilitates tumor angiogenesis (136), offering close pro-angiogenic and tissue-remodeling support to sprouting and anastomotic blood vessels.

### 6.2 DMφ is essential in spiral artery remodeling

In a successful pregnancy, angiogenesis and spiral artery remodeling of the decidua are necessary to guarantee necessary blood flow and nutrition to the placenta and fetus, which requires the joint coordination of dNK cells, EVT, dMφ, and vascular smooth muscle cells (VSMCs). Previous studies indicate that spiral arteriole remodeling in human pregnancy occurs in two phases (146). The first phase is trophoblast-independent and occurs within the spiral artery in the endometrium. In this phase, the perivascular VSMC is disrupted, the endothelium swells and loses its continuity, and VSMCs and endothelial cells are partially induced into apoptosis, which proves to be associated with the localized accumulation of dMφ (145). Here, dMφ infiltrating remodeling vessels assist in clearing apoptotic cells and producing MMP-7 and MMP-9, which facilitate perivascular ECM degradation and the vascular remodeling process (146). The second phase, namely, trophoblast-dependent, refers to the EVT migrating into the spiral arteries and forming a pseudo-endothelium with the loss of most VSMCs and endothelial cells. Here, vasoactive intestinal peptide and pregnancy-specific glycoproteins, produced by trophoblasts, disrupt endothelial cell networks and promote the growth of nascent blood vessels via an effect on macrophages (147). In both stages, the decidual spiral arteriole endothelial cells express and secrete CCL14 to induce chemotaxis of dNK and dMφ (148). Similar to tumor angiogenesis, Ang-1, Ang-2, and Tie-2, are identified to present in the developing placenta as well as EVT and endothelial cells and VSMCs of the uterine spiral arteries, resulting in the organized breakdown and remodeling of the vasculature (144). However, the exact role of Angs in dMφ recruitment or phenotypes induction has not been explored.

Clinically, abnormal angiogenesis is an important factor leading to PE, in which dysfunctional dMφ could accelerate disease progression (149). Similar to TAMs, dMφ can regulate vascular remodeling by secreting abundant angiogenic regulators, such as VEGFA (57), placental growth factor (PlGF), and their receptors FMS-like tyrosine kinase (Flt-1) (150), among others. In pregnant mice, uterine macrophages express elevated levels of iNOS and VEGF during the implantation window compared to that of non-pregnant women (151).

**Figure 4:**
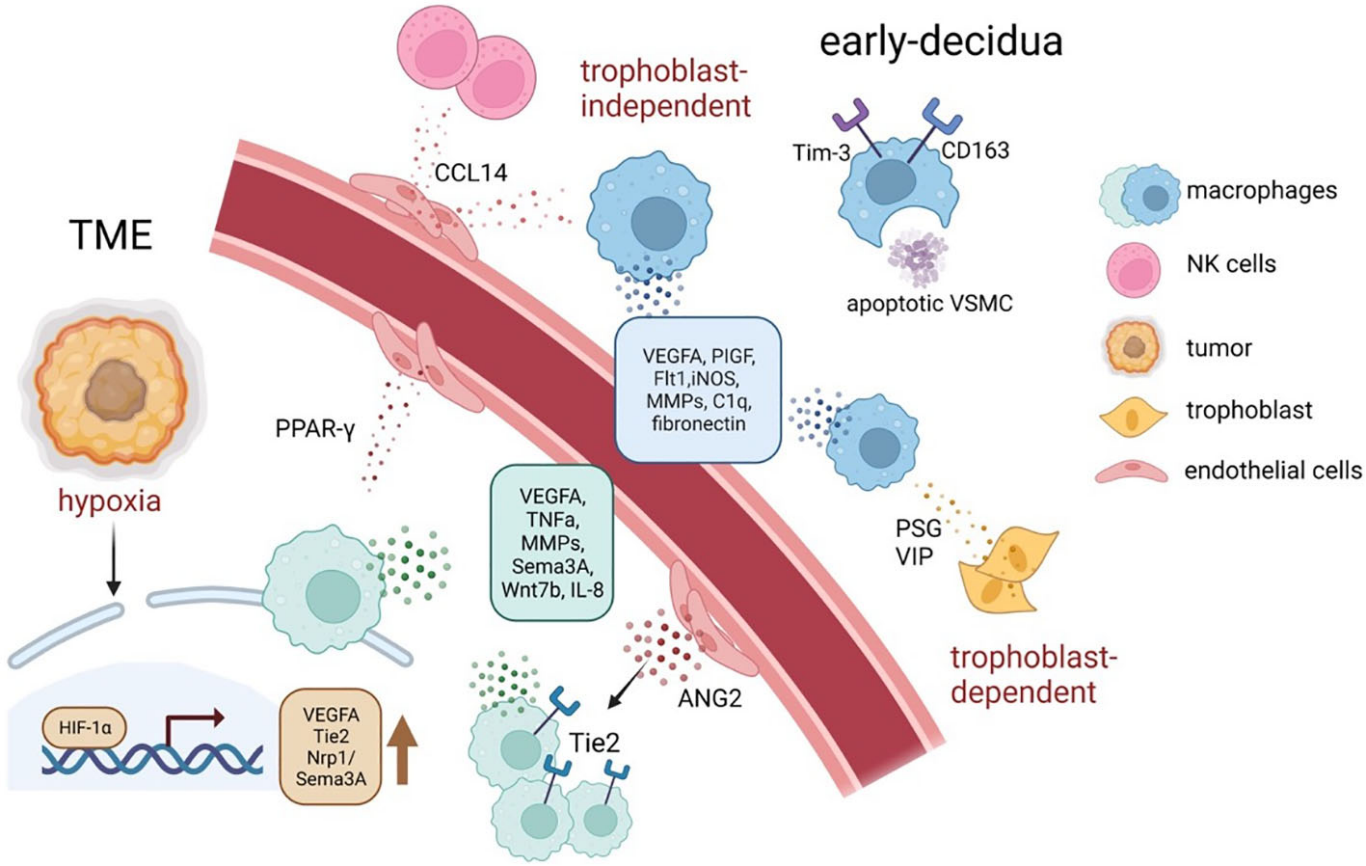
The tumor neo-angiogenesis and spiral artery remodeling during early pregnancy mediated by TAMs and dMφ. In response to distinct signals, such as hypoxia, acidosis, and high lactate, TAMs tend to accumulate in hypoxic areas of tumors and increase the secretion of a series of pro-angiogenic factors and angiogenesis-modulating factors, via upregulating HIFs (61, 145). Similarly, decidual macrophages support spiral artery remodeling by producing angiogenic factors, especially VEGF and MMPs; moreover, they maintain a homeostatic tissue environment by for instance phagocytosing apoptotic cells and debris (39). ANGPT2, angiopoietin 2; HIF, hypoxia-inducible transcription factors; MMPs, matrix metalloproteinase; TME, tumor microenvironment; VSMC, vascular smooth muscle cells; VEGFA, vascular endothelial growth factor–A.

It can be perceived that both TAM and dMφ’s regulation of angiogenesis is largely based on VEGF. Compared with studies on TAMs, studies on dMφ are limited to the mice models (152, 153), which cannot represent authentic dMφ’s dynamic change along with the human pregnancy, and, due to ethical issues and technical difficulty, it is often infeasible to acquire abundant clinical samples. However, emerging and promising technologies including organoids, microfluidics, and organ-on-a-chip are paving the way for a deeper investigation of vasculogenesis involved in placental development and tumorigenesis (154, 155). The effect of TAM and dMφ on vasculature remodeling is illustrated in **Figure 4**.

## 7 Conclusion and perspectives

The build of solid tumors is a complex multistep process requiring the interplay between different cell types and a surrounding TME to promote survival. In addition, the establishment of pregnancy adopted analogous strategies to embrace the implantation and growth of a semi-allogeneic fetus as follows (5, 8). Firstly, in terms of immunomodulation, immune tolerance is facilitated via the lack of classical MHC-I expression, presenting co-inhibitory signals and producing immunosuppressive cytokines and regulatory metabolites in both settings (156). Secondly, regarding metabolism and energy taking, both tumor and trophoblast proliferate at low oxygen levels and keep invasion toward higher oxygen levels, meanwhile releasing lactate as an energy source and signaling molecule (5). Thirdly, as mentioned before, several correlates have been drawn between tumor and pregnancy establishment in terms of angiogenesis and cell invasion, such as the upregulated level of Ang-2 and VEGFA in both settings. To maintain the growing demand for oxygen and nutrients, both tumor and trophoblast strive for the host’s blood supply via the releasing of pro-angiogenic factors and attracting various leukocytes including macrophages and NK cells (5). The above explains the similarities of macrophage phenotypes and functions in both contexts as this review has illustrated earlier.

However, there is ample evidence indicating that metabolic, proliferative, migrative, and invasive states of trophoblasts are supervised tightly and finely, in contrast to tumor cells (6, 157). For example, ordered spiral artery remodeling and confused vasculogenic mimicry as well as the well-organized invasion endpoint and distant metastases are present in the decidua and the tumor site, respectively (158). Different from the solitary tumor, ahead of embryo implantation and trophoblast presenting, the human uterus has braced itself for the fetus and finished decidualization orchestrated by various sex hormones, generating an essential nourishing and supporting population as DSCs (159). They stand opposite the trophoblast at the decidua and play an active role in directing a moderate trophoblast invasion via the secretion of a variety of different cytokines and growth factors rather than passively receiving the intrusion without any restrains, including leukemia inhibitory factor, IL-6, IL-11, IL-15, CXCL-10, and granulocyte-macrophage colony-stimulating factor (GM-CSF) (110). Also, fluctuated hormone levels at the decidua during pregnancy, especially estrogen and progesterone, affect macrophages’ phenotype and response (160). In addition, as mentioned earlier, owing to some fine-tuned structures like EVT plugs and disappearing lymphatics, placenta establishment and immune response can be modulated by the mother in a timely way, although this initiative may be taken away during tumor growth (25). Moreover, what is easily ignored is the distinction between fetus/tumor-specific antigen recognition in pregnancy and tumors. In pregnancy, it has been reported that maternal T cells become aware of the fetal allograft exclusively through “indirect” antigen presentation as the trophoblasts also express paternal MHC molecules while infiltrating T cells in tumor sites that recognize tumor-specific antigen via both “indirect” and “direct” antigen presentation (161, 162). In other words, decidual macrophages present different MHC molecules from the fetus, whereas the TAMs and tumors present the same, suggesting the slightly different functions of macrophages in terms of antigen-specific presentation and response in these two settings.

In this review, we illustrate the heterogeneity and plasticity of TAMs and dMφ and compare the maternal–fetal immune tolerance with tumor immune escape and the placental vascular remodeling with tumor neovascularization. The remarkably high plasticity and diversity allow for performing a plethora of functions with phenotype governed by the local microenvironmental milieu, which can be corroborated by both TAMs and dMφ. In general, macrophages at both the tumor sites and maternal–fetal interfaces are inclined to adopt a homeostatic M2-like phenotype, resulting in negative outcomes (tumor progression and spreading) and positive outcomes (fetus development and pregnancy maintenance), respectively. To suppress cancer, we strive to reverse the state of exhausted immune status and enhance attack by reducing anti-inflammatory TAMs and enabling pro-inflammatory TAMs. During pregnancy, dMφ provides an immunotolerant environment for the semi-allogeneic fetus to be carried in the uterus. Therefore, the switching of macrophage polarity could be a potential therapeutic target to treat pregnancy complications as is predicted for cancer treatment and vice versa. The signaling pathways and chemokine/cytokines mentioned earlier make it tantalizing to speculate that the same regulator could impact macrophage biology to aid both physiological and pathological processes. It urges researchers to make a more precise understanding of molecular mechanisms involved in macrophage function, based on animal models and clinical data, which will be crucial for developing macrophage-targeted therapeutics.

Furthermore, in addition to simple tumors or pregnancy, some particular cases should be taken into consideration as well. Decidual macrophages secrete both pro-inflammatory and anti-inflammatory cytokines, which serves as an attempt to maintain fetal tolerance while responding to microbial challenge. Take *Listeria monocytogenes*: a Gram-positive bacterium initially infiltrates placental tissues and leads to intrauterine infection, which can cause miscarriage or preterm birth (163). Upon *Listeria* infection during pregnancy, the signatures for pro-inflammatory pathways, as well as cytokine production, are strongly upregulated in dMφs (163), indicating that dangerous stimuli may disturb the balance of M1/M2 mix, reverse the dominated homeostatic dMφs, and lead to adverse outcomes. Moreover, regarding patients with cancer with pregnancy, most studies focus on the epidemiology and individual management and little discuss the dynamic phenotype and function of immune cells at decidua and concurrent tumor sites (164). In the future, whether the plasticity and polarization of local macrophages can be mutually affected by two abnormal immune statuses requires further investigations, which is quite meaningful for patients with cancer who wish to become or are pregnant.

## Author contributions

TY: Visualization, Writing – original draft, Writing – review & editing. XL: Investigation, Writing – original draft. YL: Conceptualization, Supervision, Writing – review & editing. XZ: Conceptualization, Supervision, Writing – review & editing. LL: Conceptualization, Supervision, Writing – review & editing. MD: Conceptualization, Supervision, Validation, Writing – review & editing.
